# Supplementary material for: Genetic Diversity and Genome-Wide Association Analysis of the Hulled/Naked Trait in a Barley Collection from Shanghai Agricultural Gene Bank
Source: Int J Mol Sci. 2024 May 10;25(10):5217. doi: 10.3390/ijms25105217 (PMC11120781; doi:10.3390/ijms25105217)
Supplement: Supplementary file 1 [file ijms-25-05217-s001.zip › Supplementary Tables.pdf]

Table S1. The flanking sequence of SNP 7H-523092315

Candidate SNP	The flanking sequence
7H-523092315	ACGCTGACGAGATGAAGACGTTCTTCAGGCTGTGAGTAATCCAAATTCCTTCGGCCGCAAATAATTTCCGTGATGACAATGCCAAAGCTATAAACATCAACCTTTTCGTTGATAACAGACGTCAGCCACTCTGGAGCTAAGTAACCTGGC**[G/A]**TGCCTCTCAACCGAGTCATGACACTGCTCTGCTCGCGATCTATTAGCTTGGCAAGTCCAAAGTCAGATACCTTAGCAGTGAACTGCTCATCCAAAAGGATATTTTGTGGTTTGATGTCCAGATGAGCTATTGTTTGCCGGCAGTCACTAT

Table S2. Genotyping of SNP 7H-523092315 locus by KASP and GBS respectively

Code	Genotyping by KASP	Genotyping by GBS	Hulled/naked
B01	G:G	GG	Hulled
B02	G:G	GG	Hulled
B03	G:G	GG	Hulled
B04	G:G	GG	Hulled
B05	G:G	GG	Hulled
B06	G:G	GG	Hulled
B07	G:G	GG	Hulled
B08	G:G	GG	Hulled
B09	G:G	GG	Hulled
B10	G:G	GG	Hulled
B11	G:G	GG	Hulled
B12	G:G	GG	Hulled
B13	G:G	GG	Hulled
B14	G:G	GG	Hulled
B15	G:G	GG	Hulled
B16	G:G	GG	Hulled
B17	G:G	GG	Hulled
B18	G:G	GG	Hulled
B19	G:G	GG	Hulled
B20	G:G	GG	Hulled
B21	G:G	GG	Hulled
B22	G:G	GG	Hulled
B23	G:G	GG	Hulled
B24	G:G	GG	Hulled
B25	G:G	GG	Hulled
B26	G:G	GG	Hulled
B27	Undetermined	GG	Hulled
B28	G:G	GG	Hulled
B29	G:G	GG	Hulled
B30	G:G	GG	Hulled
B31	G:G	GG	Hulled
B32	G:G	GG	Hulled

B33	G:G	GG	Hulled
B34	G:G	GG	Hulled
B35	G:G	GG	Hulled
B36	G:G	GG	Hulled
B37	G:G	GG	Hulled
B38	G:G	GG	Hulled
B39	G:G	GG	Hulled
B40	G:G	GG	Hulled
B41	G:G	GG	Hulled
B42	G:G	GG	Hulled
B43	G:G	GG	Hulled
B44	G:G	GG	Hulled
B45	G:G	GG	Hulled
B46	G:G	GG	Hulled
B47	G:G	GG	Hulled
B48	G:G	GG	Hulled
B49	G:G	GG	Hulled
B50	G:G	GG	Hulled
B51	G:G	GG	Hulled
B52	G:G	GG	Hulled
B53	G:G	GG	Hulled
B54	G:G	GG	Hulled
B55	G:G	GG	Hulled
B56	G:G	GG	Hulled
B57	G:G	GG	Hulled
B58	G:G	GG	Hulled
B59	G:G	GG	Hulled
B60	G:G	GG	Hulled
B61	G:G	GG	Hulled
B62	G:G	GG	Hulled
B63	G:G	GG	Hulled
B64	G:G	GG	Hulled
B65	G:G	GG	Hulled
B66	G:G	GG	Hulled
B67	G:G	GG	Hulled
B68	G:G	GG	Hulled
B69	G:G	GG	Hulled
B70	G:G	GG	Hulled
B71	G:G	GG	Hulled
B72	G:G	GG	Hulled
B73	G:G	GG	Hulled
B74	G:G	GG	Hulled
B75	G:G	GG	Hulled
B76	G:G	GG	Hulled

B77	A:A	AA	Naked
B78	A:A	AA	Naked
B79	A:A	AA	Naked
B80	A:A	AA	Naked
B81	A:A	AA	Naked
B82	A:A	AA	Naked
B83	G:G	GG	Naked
B84	A:A	AA	Naked
B85	A:A	AA	Naked
B86	A:A	AA	Naked
B87	A:A	AA	Naked
B88	A:A	AA	Naked
B89	A:A	AA	Naked
B90	G:G	GG	Naked
B91	G:G	AA	Naked
B92	A:A	AA	Naked
B93	A:A	AA	Naked
B94	A:A	AA	Naked
B95	G:G	GG	Naked
B96	A:A	AA	Naked
B97	A:A	AA	Naked
B98	G:G	GG	Naked
B99	A:A	AA	Naked
B100	A:A	AA	Naked
B101	A:A	AA	Naked
B102	A:A	AA	Naked
B103	A:A	AA	Naked
B104	G:G	GG	Naked
B105	A:A	AA	Naked
B106	A:A	AA	Naked
B107	A:A	AA	Naked
B108	A:A	AA	Naked
B109	A:A	AA	Naked
B110	A:A	AA	Naked
B111	G:G	GG	Hulled
B112	G:G	GG	Hulled
B113	G:G	GG	Hulled
B114	G:G	GG	Hulled
B115	A:A	AA	Naked
B116	A:A	AA	Naked
B117	A:A	AA	Naked
B118	A:A	AA	Naked
B119	A:A	AA	Naked
B120	A:A	AA	Naked

B121	A:A	AA	Naked
B122	A:A	AA	Naked
B123	G:G	GG	Hulled
B124	G:G	GG	Hulled
B125	G:G	GG	Hulled
B126	G:G	GG	Hulled
B127	G:G	GG	Hulled
B128	G:G	GG	Hulled
B129	G:G	GG	Hulled
B130	G:G	GG	Hulled
B131	G:G	GG	Hulled
B132	G:G	GG	Hulled
B133	G:G	GG	Hulled
B134	G:G	GG	Hulled
B135	G:G	GG	Hulled
B136	G:G	GG	Hulled
B137	G:G	GG	Hulled
B138	G:G	GG	Hulled
B139	G:G	GG	Hulled
B140	G:G	GG	Hulled
B141	G:G	GG	Hulled
B142	G:G	GG	Hulled
B143	G:G	GG	Hulled
B144	G:G	GG	Hulled
B145	G:G	GG	Hulled
B146	A:A	AA	Naked
B147	G:G	GG	Hulled
B148	G:G	GG	Hulled
B149	G:G	GG	Hulled
B150	G:G	GG	Hulled
B151	A:A	AA	Naked
B152	G:G	GG	Hulled
B153	G:G	GG	Hulled
B154	G:G	GG	Hulled
B155	G:G	GG	Hulled
B156	G:G	GG	Hulled
B157	G:G	GG	Hulled
B158	A:A	AA	Naked
B159	G:G	GG	Hulled
B160	G:G	GG	Hulled
B161	G:G	GG	Hulled
B162	G:G	GG	Hulled
B163	G:G	GG	Hulled
B164	G:G	GG	Hulled

B165	G:G	GG	Hulled
B166	G:G	GG	Hulled
B167	G:G	GG	Hulled
B168	G:G	GG	Hulled
B170	G:G	GG	Hulled
B171	G:G	GG	Hulled
B172	G:G	GG	Hulled
B173	G:G	GG	Hulled
B174	G:G	GG	Hulled
B175	G:G	GG	Hulled
B176	G:G	GG	Hulled
B177	G:G	GG	Hulled
B180	G:G	GG	Hulled
B181	G:G	GG	Hulled
B182	G:G	GG	Hulled
B183	G:G	GG	Hulled
hua30	G:G	GG	Hulled
hua22	G:G	GG	Hulled
hua11	G:G	GG	Hulled

Tale S3. Summary of DNA-seq data from seedlings of different barley germplasms

Samples	Raw Base(G)	Clean Base (G)	GC Content (%)	Clean Q30 (%)
B01	1.64	1.57	47.88	90.60
B02	1.57	1.51	48.45	92.03
B03	1.66	1.60	48.70	91.16
B04	1.58	1.52	48.33	91.14
B05	1.73	1.66	48.32	90.92
B06	1.60	1.52	47.80	90.30
B07	1.62	1.56	48.16	90.99
B08	1.55	1.49	48.14	91.17
B09	1.67	1.61	48.19	91.44
B10	1.72	1.65	48.04	90.78
B11	1.66	1.61	48.28	91.83
B12	1.67	1.61	48.55	92.02
B13	1.68	1.62	48.05	91.34
B14	1.63	1.57	48.15	91.10
B15	1.66	1.60	47.92	90.87
B16	1.59	1.54	48.34	91.74
B17	1.66	1.60	48.11	90.89
B18	1.60	1.54	47.36	90.27
B19	1.65	1.59	47.90	91.10
B20	1.61	1.56	47.73	91.19
B21	1.59	1.53	48.59	90.25
B22	1.61	1.56	48.28	90.58

B23	1.83	1.77	48.50	91.70
B24	1.68	1.62	48.17	90.91
B25	1.66	1.60	48.15	91.00
B26	1.59	1.53	48.56	91.28
B27	1.64	1.58	48.06	91.10
B28	1.65	1.60	48.24	91.11
B29	3.79	3.66	48.00	91.27
B30	1.57	1.51	48.5	91.22
B31	1.59	1.53	48.83	91.69
B32	1.56	1.51	48.32	91.04
B33	1.53	1.48	48.42	91.13
B34	1.54	1.48	48.47	91.28
B35	1.52	1.45	48.47	90.65
B36	1.65	1.59	48.16	91.13
B37	1.57	1.52	48.35	91.14
B38	1.53	1.47	48.29	90.99
B39	1.67	1.61	47.95	91.15
B40	1.71	1.66	48.70	91.45
B41	1.73	1.66	48.58	91.58
B42	1.67	1.61	48.60	91.73
B43	1.69	1.63	48.81	92.32
B44	1.67	1.61	48.67	91.89
B45	1.72	1.66	48.57	91.90
B46	1.65	1.59	48.56	91.58
B47	1.49	1.43	48.01	91.15
B48	1.76	1.69	48.60	91.99
B49	1.64	1.56	48.62	90.56
B50	1.55	1.47	48.91	90.57
B51	1.51	1.43	48.53	89.97
B52	1.60	1.53	48.80	90.72
B53	1.60	1.52	48.37	90.30
B54	1.57	1.49	48.32	90.27
B55	1.53	1.46	48.99	90.62
B56	1.60	1.52	48.51	90.66
B57	1.58	1.49	48.33	89.61
B58	1.34	1.24	48.67	88.20
B59	1.49	1.41	48.68	89.91
B60	1.52	1.43	48.56	89.90
B61	1.46	1.37	48.32	89.19
B62	1.45	1.39	48.53	90.33
B63	1.51	1.44	48.63	90.33
B64	1.51	1.43	48.20	90.02
B65	1.53	1.47	48.35	90.61
B66	1.53	1.46	48.57	90.49

B67	1.55	1.48	48.80	90.17
B68	1.57	1.50	48.54	90.42
B69	1.52	1.46	48.43	90.27
B70	1.49	1.42	49.20	90.87
B71	1.62	1.55	48.84	90.84
B72	1.56	1.49	48.93	90.92
B73	1.46	1.39	48.02	88.93
B74	1.40	1.32	48.39	89.16
B75	1.40	1.32	48.31	88.49
B76	1.34	1.26	48.27	88.65
B77	1.48	1.41	47.96	89.47
B78	1.43	1.35	47.90	88.75
B79	1.40	1.32	48.08	88.41
B80	1.44	1.36	48.45	88.86
B81	1.40	1.35	48.84	91.07
B82	1.50	1.43	48.54	90.58
B83	1.45	1.39	48.66	90.82
B84	1.47	1.41	48.84	90.68
B85	1.47	1.41	48.74	90.60
B86	1.48	1.42	48.71	90.88
B87	1.45	1.38	48.89	90.60
B88	1.45	1.38	48.57	90.25
B89	1.50	1.41	47.38	88.38
B90	1.42	1.35	47.07	88.90
B91	1.42	1.34	47.78	88.57
B92	1.47	1.40	48.10	89.86
B93	1.57	1.49	47.73	89.46
B94	1.48	1.40	47.75	88.83
B95	1.41	1.32	47.07	88.55
B96	1.53	1.45	47.46	88.23
B97	1.36	1.29	48.01	89.48
B98	1.40	1.33	47.84	89.49
B99	1.39	1.32	48.04	89.70
B100	1.35	1.28	48.07	89.29
B101	1.36	1.29	48.23	89.72
B102	1.30	1.24	47.66	89.25
B103	1.42	1.35	47.67	89.64
B104	1.39	1.32	48.02	89.48
B105	1.45	1.38	47.53	89.06
B106	1.45	1.38	48.04	89.84
B107	1.47	1.39	48.27	88.96
B108	1.50	1.42	48.07	89.89
B109	1.39	1.32	47.84	89.87
B110	1.40	1.33	47.99	89.51

B111	1.51	1.44	47.91	89.42
B112	1.41	1.34	48.23	89.57
B113	1.51	1.44	47.99	90.65
B114	1.44	1.36	48.47	90.66
B115	1.52	1.45	48.14	91.14
B116	1.48	1.40	48.43	91.31
B117	1.52	1.43	48.59	91.47
B118	1.62	1.53	48.34	91.17
B119	1.37	1.30	48.29	91.04
B120	1.62	1.53	48.70	91.66
B121	1.43	1.37	47.76	91.05
B122	1.40	1.33	48.54	90.67
B123	1.33	1.27	48.19	90.69
B124	1.55	1.49	48.13	91.40
B125	1.70	1.62	47.96	91.38
B126	1.52	1.45	47.46	90.77
B127	1.66	1.59	47.85	91.67
B128	1.53	1.47	47.73	91.40
B129	1.40	1.34	48.08	90.73
B130	1.32	1.25	48.29	89.64
B131	1.58	1.51	47.97	91.13
B132	1.47	1.41	47.57	90.36
B133	1.26	1.20	48.37	90.16
B134	1.46	1.41	47.41	90.66
B135	1.32	1.28	46.96	91.13
B136	1.28	1.22	47.19	89.62
B137	1.43	1.38	47.30	90.72
B138	1.33	1.28	47.37	90.38
B139	1.29	1.23	47.27	90.20
B140	1.23	1.17	47.68	89.82
B141	1.33	1.28	47.85	90.88
B142	1.09	1.04	47.49	89.61
B143	1.48	1.43	48.11	91.42
B144	1.38	1.33	47.18	90.88
B145	1.19	1.14	47.58	90.24
B146	1.45	1.40	47.12	90.56
B147	1.52	1.46	47.81	91.21
B148	1.41	1.36	48.99	91.32
B149	1.44	1.39	48.08	91.13
B150	1.36	1.30	47.42	90.37
B151	1.50	1.44	47.54	90.86
B152	1.43	1.38	46.82	90.26
B153	1.41	1.36	47.08	90.99
B154	1.48	1.43	48.51	91.42

B155	1.41	1.35	48.32	91.24
B156	1.61	1.54	48.48	91.83
B157	1.53	1.47	48.17	91.60
B158	1.66	1.60	48.27	92.39
B159	1.51	1.45	48.03	91.61
B160	1.59	1.54	49.28	92.56
B161	1.20	1.14	47.67	89.37
B162	1.32	1.26	47.38	89.83
B163	1.30	1.23	47.40	89.22
B164	1.29	1.23	47.31	90.00
B165	1.22	1.15	48.30	89.76
B166	1.18	1.11	47.58	89.45
B167	1.21	1.14	47.02	89.90
B168	1.28	1.20	47.03	89.40
B170	1.46	1.40	47.87	91.28
B171	1.52	1.47	47.49	91.30
B172	1.53	1.48	47.70	91.26
B173	1.50	1.44	47.71	91.15
B174	1.54	1.48	48.22	91.17
B175	1.55	1.49	48.08	91.46
B176	1.61	1.54	48.13	91.30
B177	1.47	1.41	47.96	91.00
B180	1.21	1.17	46.44	89.77
B181	1.31	1.27	48.26	91.85
B182	1.41	1.34	48.52	90.05
B183	1.57	1.51	47.42	90.40
hua11	1.33	1.29	47.59	90.72
hua22	1.53	1.47	47.72	89.91
hua30	1.37	1.31	47.50	90.49
